# Diagnosing the silent: the molecular landscape of non-functional parathyroid carcinoma

**DOI:** 10.1007/s00428-025-04193-4

**Published:** 2025-08-05

**Authors:** Maaia Margo Jentus, Willem E. Corver, Marieke Snel, Femke M. van Haalen, Tom van Wezel, Dina Ruano, Ellen Kapiteijn, Stijn Crobach, Natasha M. Appelman-Dijkstra, Abbey Schepers, Hans Morreau

**Affiliations:** 1 Department of Pathology, Leiden University Medical Center, Leiden, The Netherlands; 2 Department of Medicine, Division of Endocrinology, Leiden University Medical Center, Leiden, The Netherlands; 3 Department of Surgery, Leiden University Medical Center, Leiden, The Netherlands; 4 Department of Medical Oncology, Leiden University Medical Center, Leiden, The Netherlands

**Keywords:** Parathyroid carcinoma, Non-functional parathyroid carcinoma, Parathyroid, Whole chromosome loss, Molecular analysis, Endocrine pathology, Parathyroid neoplasia, Rare cancer

## Abstract

Non-functional parathyroid carcinoma (NFPC) is an exceptionally rare malignancy, with approximately 50 cases reported since the parathyroid glands were first described in the late nineteenth century. Unlike functional parathyroid carcinomas (PC), which typically present with severe hyperparathyroidism (HPT) and are monitored via serum calcium and parathyroid hormone (PTH) levels, NFPC lacks biochemical markers and is monitored solely through imaging. In this study, we present two new cases of primary NFPC and provide a comprehensive review of the literature, including a detailed analysis of immunohistochemical and molecular testing. We also explore potential mechanisms underlying the non-functional state in PC. In one case, the tumor lacked PTH expression by immunohistochemistry, yet low levels of *PTH* mRNA expression were consistently detected. Differential diagnoses—including medullary and non-medullary thyroid carcinoma, paraganglioma, and other head and neck tumors—were excluded. The second case demonstrated patchy residual PTH immunostaining. Both tumors exhibited extensive whole chromosome losses resulting in a near-haploid genome, with (NFPC 2) or without (NFPC 1) subsequent endoreduplication/genome doubling. The mutational landscape differed between the two, with neither showing *CDC73* mutations or loss of parafibromin expression. Diagnosing primary non-functional parathyroid carcinoma can be especially challenging in cases with negative PTH immunohistochemistry.

**Supplementary Information:**

The online version contains supplementary material available at 10.1007/s00428-025-04193-4.

## Introduction

Parathyroid carcinoma (PC) is a very rare malignancy, typically suspected based on clinical features such as markedly elevated serum calcium and parathyroid hormone (PTH) levels, osteoporosis, a palpable neck mass, and a higher prevalence in male patients [1–4]. Among PC cases, non-functional PC (NFPC) is exceptionally rare, with approximately 50 cases reported to date (Table 1).

**Table 1:** Reported cases of nonfunctional parathyroid carcinoma and diagnostical milestones

Milestone	Report: author, year	Patient						Immunohistochemistry						Molecular findings
		Sex	Age	Serum Ca, mmol/L (2.12–2.55*)	Serum PTH, pmol/L (0.7–8 pmol/L)		Tumor size, cm	PTH	Parafibromin	Other positive	Other weak/patchy	Other negative	Ki67/MIB1	
Description of the parathyroid glands by Sandstrom (1880)														
First reported PC	De Quervain [5]	m	68				6.5	XXX						
	Roffo [6]	m	60				Ostrich egg	XXX						
Normal reference for human serum Calcium by Howland and Marriott (1918)														
Protocol and introduction of blood Calcium assay by Kramer and Tisdall (1921)														
Relation of parathyroid with Calcium by Salvesen (1923)														
Discovery of parathyroid hormone by Collip (1925)														
Formal description of hyperparathyroidism by Mandl (1925)														
	Toland [7]	f	60				na	XXX						
	Price and Movat [8]	m	49				Hen’s egg	XXX						
	Hall and Chaffin [ 9]	m	46	2.69			11	XXX						
	McQuillan [10]	f	53	2.67			Golf ball	Diagnosis based on metastasis						
Labeling antibodies with fluorescent dyes — basis of IHC by Coons (1941)														
	Sieracki and Horn [ 11]	f	43	2.27			3.5	XXX						
	Pachter and Lattes [12]	f	50	N			na	XXX						
PTH RAI (radioimmunoassay) by Yalow and Bernson (1963)														
	Altenähr and Saeger [13]	m	50	N		na	na	XXX						
	Farr et al. [14]	na	na	Retrospective study of 100 parathyroid tumors of a single center, one normocalcemic (“non-functional”) carcinoma										
Introduction of monoclonal antibodies with following widely adaptation by Köhler and Milstein (1957)														
Sanger Sequencing by Sanger (1977)														
Amino acid sequencing of human PTH by Keutmann (1978)														
	Dhom and Hohbach [15]	m	38	na		na	2.5	XXX						na
	Chahinian et al. [16]	f	69	2.29		N	14	XXX						na
	Aldinger et al. [17]	m	27	2.45		na	na	XXX						na
	Aldinger et al. [17]	m	49	N		na	na	XXX						na
	Ordoñez et al. [18]	m	27	2.3		na	5.5	+		na	na	na	na	na
	Ordoñez et al. [18]	m	59	2.4		na	2.5	+		na	na	na	na	na
Invention of PCR by Mullis (1983)														
Sequencing of *PTH* by Vasicek (1983)														
	Yamashita et al. [19]	f	69	2.29		na	5	na		na	na	TG	na	na
	Merlano et al. [20]	m	59	N		N	8	na		na	na	na	na	na
	Collins et al. [21]	m	65	2.34		na	11	na		na	na	na	na	na
	Murphy et al. [22]	m	51	N		N	n/a	+ -		na	CgA, NSE	na	na	na
	Yamashita et al. [23]	f	56	2.32		na	3	- +		CgA	na	JT1, CT, TG	na	na
	Eurelings et al. [24]	f	45	N		na	na	-		CgA, Vim	na	CK NOS	na	na
	Jung et al. [25]	m	40	N		N	na	na		na	na	CT	na	na
	Im et al. [26]	m	60	N		N	na	na		na	na	na	na	na
Parafibromin staining as marker of CDC73 status in parathyroid carcinoma by Tan (2004)														
	Kirkby-Bott et al. [27]	m	66	N		na	na	“IHC showed a poorly differentiated endocrine tumor of parathyroid origin”						na
Invention of the next generation sequencing (NGS) by McCooke (2006)														
	Ashkenazi et al. [28]	f	58	N		6.53	na	na	na	na	na	na	na	na
	Grodski et al. [29]	m	46	2.35		na	12	1%	na	na	na	CT, TG, TTF1	na	Negative family history, pending results of HPT-JT and MEN1 genetic screening
	Mazeh et al. [30]	f	44	2.48		na	1.5	na	na	na	na	na	na	na
	Wilkins and Lewis [31]	m	59	2.52		2.1	6	+	na	CK AE1/AE3	CD56, CgA, Syp	CD5, CD45, CT, CEA, TG, TTF1	na	na
	Choi [32]	m	44	1.23		1.0	2,2	+	na	CgA, Syp	na	CD34, CD45, CT, TG, TTF1	“weak”	na
	Gao et al. [33]	m	47	2.63		4.5	4	50% (+)	na	na	na	CT, “Neuropeptides,” TG	15%	na
	Nakamura et al. [34]	f	65	2.19		3.9	5	-	na	CgA, CycD1, NSE	na	CA19-9, CEA, CT EMA, TG, S100	na	na
	Kotromanović et al. [35]	m	65	2.28		5.0	8	na	na	NSE	na	CT, CgA, NF, TG	na	na
	Piciu et al. [36]	f	51	2.27		5.7	2.5	+ -	na	CK AE1/AE3, NSE	CK7, CgA, GCDFP15, PR	CT, CD56, CK20, ER, MG, Syp, TG	70%	na
	Posada-González et al. [37]	m	54	2.3		6.1	1.4	na	na	CycD1	na	na	na	MEN2a patient, heterozygous mutation (Cys618Arg) in exon 10 of *RET*
	Cetani et al. [ 38]	m	50	2.42		7.16	1.3	+	na	CgA	na	TG	na	na
	Wang et al. [39]	f	49	2.33		4.2	1	na	na	PanCK, CgA, Syp	na	BCL2, CEA, CT, HMB45, S100, TG, TTF1	58%	na
	Suganuma et al. [40]	m	67	N		5.2	4.2	+ -	na	na	CgA	CD5, CT, S100, TG, TTF1	5%	RT-PCR for PTH mRNA
	Bartoňová et al. [41]	m	39	2.25		3.1	na	-	na	Galectin 3	CgA, CycD1	CEA, Syp, TG, TTF1	10%	na
	Khalil et al. [42]	m	46	na		na	7,5	- +	partial loss	CgA, GATA3, Vim	Cam5.2, PanCK, Syp	CT, CD56, p63, PAX8, SMA, SST, S100, TTF1	na	An activating mutation in *PIK3CA* and a frameshift mutation in *MEN1*
	Sen et al. [43]	m	42	2.42		6.6	4	+	+	na	na	CgA, TG, TTF1	10%	na
	Šplíchalová et al. [44]	f	26	N		N	na	na	na	na	na	na	na	na
	Abdullah et al. [45]	m	59	N		na	na	na	na	na	na	na	na	na
	Ivaniš et al. [46]	f	47	2.4		4.4	1	+	na	na	na	CgA, TTF1	na	na
	Mremi et al. [47]	m	42	N		N	12	+	na	na	na	na	na	na
	Yin et al. [48]	f	54	2.2		6.0	1	+ -	na	CD117, CK AE1/AE3, GATA3	CgA, Syp	CT, TG, PAX8	< 5%	na
	Jentus et al. 2025—this study	f	64	2.33		6.1	14	-	+	GATA3, SDHA, SDHB	CK AE1/AE3	CT, S100, TTF1, PAX8	2–3%	*TERT*p c.−124C > T *MEN1* c.1116delA, p. (Ala373Profs*9) Amplification of *CCND3* (17 copies), low TMB (1.9 mutations/Mb), MSS. LOH of chr. 1, 2, 3, 6, 8, 9, 10, 11, 12, 13, 15, 17, 18 and 22. No gene fusions detected
	Jentus et al. 2025—this study	f	65	2.31		5.2	2.4	+	+	CgA	na	TTF1	30%	*PTEN* c.1003C > T, p.(Arg335*) *ARID1B* c.954_956delAGG, p.(Gly319del) SETD2 c.5122C > T, p.(Arg1708*) KDM5C c.2080C > T, p.(Arg694*) Low TMB (11.46 mutations/Mb) MSS cnLOH of chr. 1, 2, 3, 4, 8, 10, 11, 12, 13, 15, 17, 18, 21, and 22; heterozygous chr. 5, 6, 7, 9, 14,16, 19, and 20 with “balanced gain”

^*^Critical upper value for Calcium > 3.25 mmol/L

XXX histology-based diagnosis, no symptoms of hyperparathyroidism, clinical and/or radiological investigations without abnormalities

*CK* cytokeratin, *chr.* chromosome(s), *cnLOH* copy neutral loss of heterozygosity, *CT* calcitonin, *CycD1* cyclin D1, *EM* electron microscopy, *HPT* hyperparathyroidism, *IHC* immunohistochemistry, *MG* mammaglobin, *MSS* microsatellite stable, *na* not available, *N* normal, *NF* neurofilament, *NGS* next generation sequencing, *RT-PCR* reverse transcription polymerase chain reaction, *SST* somatostatin, *Syp* synaptophysin, *Vim* vimentin

NFPC is often discovered incidentally during the evaluation of suspected thyroid neoplasms—particularly medullary thyroid carcinoma—in the absence of primary hyperparathyroidism (HPT) [41, 42]. The molecular landscape of PC has recently been characterized, with a particular focus on the *CDC73* gene, which encodes parafibromin and plays a critical role in both hereditary and sporadic PC [49–52]. Although *MEN1* mutations are a major driver in benign parathyroid neoplasms, dysfunctional *MEN1* has also occasionally been reported in PC [53–58].

In contrast, much less is known about the molecular features of NFPC. Potential mechanisms for the non-functional state include inactivation of the *PTH* gene or transcriptional downregulation. While a few mutations in the *PTH* gene have been described, these are typically associated with hypoparathyroidism and have unclear relevance to malignancy [59]. One case of a parathyroid adenoma was negative for PTH in immunohistochemistry (IHC) and produced biologically active but truncated PTH variant that could not be detected using conventional assays [60].

Patients with functional PC are typically followed up by serum PTH and calcium levels. In contrast, NFPC follow-up relies entirely on imaging due to the absence of biochemical markers [3, 61]. Preoperative recognition of a possible parathyroid origin is crucial, as intraoperative tumor spill is a significant predictor of recurrence and PC-specific mortality, whereas lymph node status appears to have no impact on overall survival [3]. Decreased mortality of PC has been reported when surgery is performed in expertise centers for endocrine surgery [3].

Previous reviews suggest that NFPC is frequently diagnosed at advanced stages, with local invasion present in 93% of cases and distant dissemination in up to 59%, compared to 30% in functional PC [46].

In this study, we reviewed previously reported NFPC cases with a focus on pathological findings, particularly IHC panels used to determine tissue origin, and available molecular data. We also report two new cases of primary NFPC, with distinctive molecular profiles.

## Materials and methods

Detailed methods are provided in Online Resource 1. The two patients reported in this study underwent surgery at our tertiary referral center for parathyroid disease. Pathology reports were reviewed retrospectively. In addition, we reassessed hematoxylin and eosin (H&E)-stained slides, immunohistochemistry (IHC) and molecular diagnostic tests (see: https://www.palga.nl/voor-pathologen/moleculaire-bepaling under “LUMC”).

For genome-wide loss of heterozygosity (GW-LOH) panel testing and subsequent Imbalance-LOH copy number variation (CNV) analysis, a 1500 single-nucleotide polymorphisms (SNP) panel was used, as previously described [62].

For NFPC Case 1 (which lacked PTH expression by IHC), fresh frozen tumor tissue was available. Frozen samples from two functional parathyroid adenomas and one primary and one metastatic functional PC were used as positive controls for PTH mRNA real-time PCR analysis. Two non-parathyroid cell lines were included as negative controls. PTH mRNA expression was assessed by real-time PCR, as previously described [40, 63]. Frozen tissue from a metastasis of NFPC Case 2 (which retained positive PTH IHC but lacked biochemical evidence of function) was also available.

For both cases, *PTH* exons 1, 2, and 3, along with flanking intronic and promoter regions, were amplified using M13-tailed primers and analyzed by Sanger sequencing.

## Results

### Case 1

A 64-year-old female patient was referred to our center with a rapidly enlarging 10-cm cervical mass causing progressive airway obstruction. There were no signs of HPT; serum calcium, phosphorus, and PTH levels were within normal limits.

Pre-operative fine-needle aspiration (FNA) of the tumor, performed at an external institution, was interpreted as a follicular thyroid neoplasm (Bethesda category IV). No cell block was available for IHC analysis to distinguish between follicular or medullary thyroid carcinoma and a parathyroid neoplasm. Surgical resection yielded three tumor fragments measuring up to 14 cm, 8 cm, and 1,5 cm in greatest dimension.

**Fig. 1:**
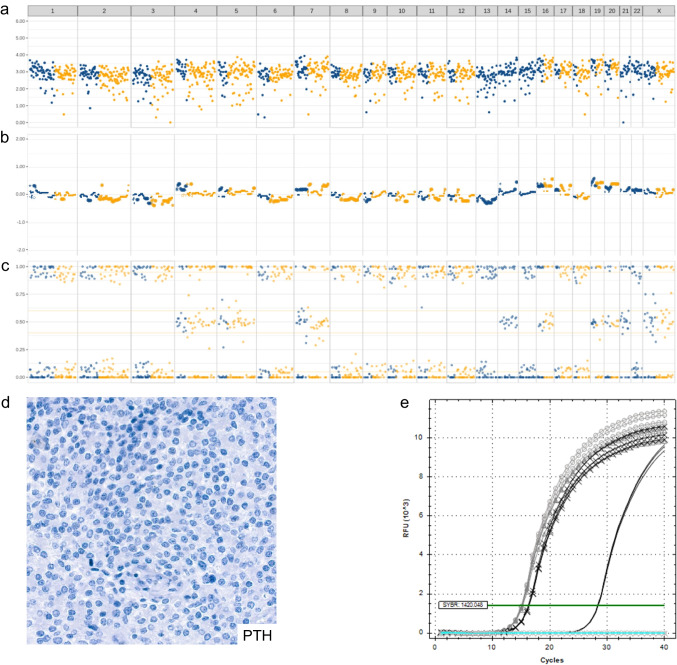
NFPC case 1. **a**–**c** Genome-wide LOH (GW-LOH) testing and Imbalance-LOH-CNV analysis (1500 single-nucleotide polymorphisms (SNP) panel). The horizontal axis spans chromosomes 1 through X; SNPs from the Y chromosome are not included. **a** Median amplicon read count (log_10_) is plotted on the vertical axis, reflecting the coverage across all 1500 SNPs. **b** Normalized median read count (log_10_), showing copy number (CN) profiles. **c** Variant allele frequency plotted for each SNP. See also the “Materials and Methods” section and Online Resource 1 for scoring criteria. Analysis of NFPC Case 1 revealed massive chromosomal losses with LOH of chromosomes 1, 2, 3, 6, 8, 9, 10, 11, 12, 13, 15, 17, 18, and 22, consistent with a near-haploid genome. OCAplus (large gene panel) analysis confirmed a similar chromosomal pattern (not shown). No evidence of endoreduplication/genome doubling was observed, as OCAplus estimated ploidy at 1.07. **d** Absence of PTH expression by immunohistochemistry (external positive control not shown). **e** PTH mRNA expression by real-time PCR. Black solid line: NFPC (case 1); gray solid lines: two functional parathyroid adenomas (PAs; positive controls); black line with crosses: a functional PC (positive control); blue solid line: water (negative control); pale pink and gray solid lines: thyroid and retina cell lines, respectively (additional negative controls, overlapping with water control and thus not distinctly visible). Mean quantification cycle (Cq) values from triplicate analysis: PAs 15.12 (SD 0.08), functional PC 16.31 (SD 0.06), NFPC 28.39 (SD 0.04). There was no significant difference in PTH Cq between the two PAs. However, Cq values differed significantly between each PA and the functional PC (*p* < 0.0001, two-tailed unpaired *t*-test). NFPC PTH Cq values were significantly higher compared to both PAs and functional PC controls (*p* < 0.0001, two-tailed unpaired *t*-test)

Histopathological examination revealed a proliferation of atypical parathyroid chief cells. However, confirmation of parathyroid origin was initially inconclusive due to negative PTH staining by IHC (Fig. 1d).

**Fig. 2:**
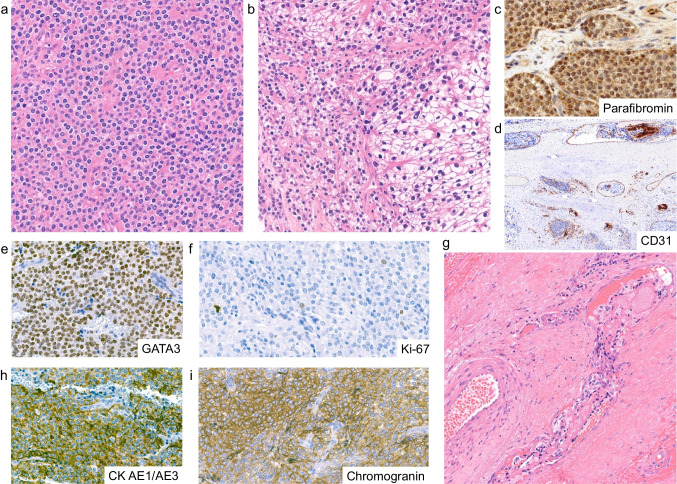
Morphological and immunohistochemical features of NFPC Case 1. **a** Sheets of monotonous tumor cells with stroma depleted of fat tissue. A mitotic figure is visible in the upper right. **b** Area showing marked cytonuclear atypia. **c** Retained nuclear parafibromin staining, suggestive of a wild-type *CDC73*. **d** Tumor cells within a blood vessel, delineated by CD31-positive endothelium. **e** Strong nuclear expression of GATA3 in tumor cells. **f** Tripolar mitotic figure captured in the background of relatively low Ki-67 proliferation index. **g** One of multiple tumor thrombi observed. **h** Patchy and heterogeneous cytoplasmatic staining for cytokeratin (CK) AE1/AE3. **i** Moderately strong, heterogeneous cytoplasmatic expression of chromogranin

Positive GATA3 IHC (Fig. 2e) supported a possible parathyroid origin. Negative staining for TTF1 and calcitonin (not shown) excluded a differentiated (follicular or medullary) thyroid neoplasm. Paraganglioma was excluded based on patchy pan-cytokeratin expression (Fig. 2h), negative synaptophysin, and absence of S100-positive sustentacular cells (not shown). Parafibromin expression was retained in the nucleus (Fig. 2c). The Ki-67 proliferation index ranged from 2 to 3%. Mitotic figures were occasionally observed. Suspected vascular invasion (Fig. 2g) was confirmed by CD31 IHC **(**Fig. 2d), while lymphovascular space invasion (LVSI) was excluded by D2-40 staining (not shown).

We performed real-time PCR analysis to assess PTH mRNA expression. Repeated testing revealed low but detectable PTH mRNA levels in tumor tissue, with a cycle threshold (Ct) value of expression that was seen in tumor material in the real-time PCR assay with a Ct value of 28.4 (Fig. 1e). In contrast, Ct values in positive controls ranged between 14 and 16, consistent with high expression levels (lower Ct values indicate higher transcript abundance). Despite the low expression, the detection of PTH mRNA supported a parathyroid origin of the tumor. All negative controls (water, thyroid, and retina cell lines) showed no amplification.

Comprehensive molecular testing of the tumor was performed using multiple next-generation sequencing (NGS) platforms (RCPL, OCAplus, GW-LOH v2., and Archer FusionPlex PAN Solid v2.0). The analysis revealed a *TERT* promoter mutation c.−124C > T and a *MEN1* c.1116delA, p. (Ala373Profs*9) mutation with accompanying loss of heterozygosity (LOH). The patient did not exhibit clinical features suggestive of MEN1 syndrome, and due to her relatively advanced age at presentation, she was not referred to clinical genetics.

The tumor also demonstrated amplification of *CCND3* (17 copies), a low tumor mutational burden (TMB) of 1.9 mutations/Mb, a microsatellite stable (MSS) phenotype, and massive whole-chromosomal losses with LOH involving chromosomes 1, 2, 3, 6, 8, 9, 10, 11, 12, 13, 15, 17, 18, and 22 (Fig. 1a), consistent with a near-haploid genome. No subsequent endoreduplication/genome doubling was detected, as the estimated ploidy was 1.07 based on OCAplus data. No gene fusions were identified. Additional sequencing of *PTH* in the tumor revealed no somatic mutations. However, several rare homozygous SNPs were detected (Online Resource 2), likely resulting from widespread chromosomal loss, including chromosome 11.

Five months after the initial surgery, the patient presented with multiple lung metastases and local recurrence, both of which were confirmed histopathologically. Serological and clinical evaluations continued to show no signs of HPT. Six months later, the patient developed progressive local disease and slow growth of the pulmonary lesions. Temozolomide treatment was initiated. While the lung nodules stabilized under therapy, the local disease continued to progress. The patient subsequently underwent radiotherapy, receiving a total dose of 50 Gy (16 fractions of 3.125 Gy, four times a week). The patient remains under follow-up with ongoing disease progression. No further chemotherapy had been initiated to date, in accordance with the patient’s preference.

### Case 2

**Fig. 3:**
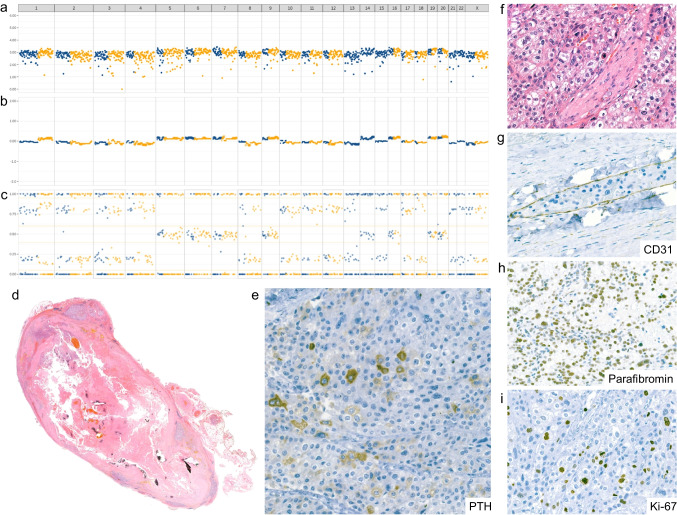
Histological and molecular findings of NFPC Case 2. **a**–**c** GW-LOH testing and Imbalance-LOH-CNV analysis (1500 SNP panel). Analysis revealed extensive chromosomal losses with LOH involving chromosomes 1, 2, 3, 4, 8, 10, 11, 12, 13, 15, 17, 18, 21, and 22, accompanied by endoreduplication/genome doubling, as indicated by extrapolated homozygous genotypes AA or BB. Heterozygous chromosomes 5, 6, 7, 9, 14,16, 19, and 20 showed extrapolated genotypes AABB. A similar chromosomal pattern was observed in OCAplus analysis (not shown). **d** Histologic section of the tumor (magnification × 7) showing central necrosis and hemorrhage. **e** PTH immunohistochemistry showing weak, patchy cytoplasmic staining. **f** Evidence of perineurial growth. **g** Vascular space within the (pseudo)capsule lined by CD31-positive endothelium containing intravascular tumor cells. **h** Retained nuclear expression of parafibromin. **i** Elevated Ki-67 proliferation index

The second patient was a 63-year-old female with no clinical or biochemical signs of HPT. She initially underwent surgery at an external institution for a 2.4-cm cervical mass. The diagnosis of primary NFPC was established based on histomorphology and positive PTH immunohistochemistry (Fig. 3).

Two years later, the patient presented with 1.9 cm paratracheal local recurrence, which was surgically removed at our institution. The recurrent tumor showed weak and patchy, but retained immunohistochemical PTH expression.

Molecular analysis using the OCAplus panel identified several alterations: a *PTEN* mutation c.1003C > T, p.(Arg335*); an *ARID1B* mutation c.954_956delAGG, p.(Gly319del); a *SETD2* mutation c.5122C > T, p.(Arg1708*); and a *KDM5C* mutation c.2080C > T, p.(Arg694*). All four genes also exhibited LOH.

The patient and family were not clinically suspect for PTEN hamartoma tumor syndrome (Cowden syndrome). Due to the patient’s age at tumor presentation, no referral to clinical genetics was made.

The tumor had a low TMB (11.46 mutations/Mb), was MMR proficient, and showed extensive whole chromosome losses involving chromosomes 1, 2, 3, 4, 8, 10, 11, 12, 13, 15, 17, 18, 21, and 22, with extrapolated genotypes of AA or BB, indicating endoreduplication/genome doubling. Heterozygous chromosomes 5, 6, 7, 9, 14,16, 19, and 20 showed extrapolated AABB genotypes. The endoreduplication/genome doubling was supported by an estimated ploidy of 2.45 in the OCAplus analysis.

Additional sequencing of *PTH* revealed no somatic mutations. As in Case 1, several rare homozygous *PTH* SNP alleles in *PTH* were identified, likely resulting from extensive chromosomal loss, including chromosome 11. As of 2 years after recurrence, the patient shows no evidence of disease.

## Review of previously reported NFPC

We reviewed previously published cases of NFPC, focusing on the pathological workup, including immunohistochemical and molecular analyses (Table 1). Pathological and clinical data from 44 cases were compatible with NFPC, to which we now add two additional cases, bringing the total to 46. Several articles could not be obtained for the review, though their titles suggest they may describe at least four additional NFPC cases [64–72].

The included literature spans a range of diagnostic eras, as reflected by the evolution of available techniques (milestones) at the time of each report. Detailed clinical parameters were not the scope of this review. For a comprehensive clinical overview, we refer readers to the recent work by Ivanis et al. [46]. Our review instead focused on the biological and histopathological features of NFPC, aiming to identify diagnostic clues and potential pitfalls relevant to modern diagnostic practice.

Histopathological criteria for the diagnosis of PC, as proposed by Castleman and Schantz and incorporated into the WHO Classification of Tumours, have often been referenced in the literature. However, these criteria primarily aid in distinguishing malignant from benign parathyroid disease [73, 74]. Interestingly, the first reported case of PC was a NFPC [5]. At the time, the diagnosis was solely based on histological features, and no clinical signs of hyperparathyroidism were described. However, it is important to note that the clinical entity of hyperparathyroidism had not yet been formally recognized at that time [75]. With the implementation of routine assessment of serum calcium, phosphorus, and later PTH measurements in the 1970 s, the diagnosis of NFPC became more reliable. The advent of immunohistochemistry and molecular techniques confirmed the existence of this rare entity.

As PTH levels in functional PC are typically elevated 3–10 times above the upper limit of normal, some cases with only mildly increased calcium or PTH levels were historically misclassified as non-functional [3, 4, 76]. Labeling a PC as non-functional based solely on the absence of clinical symptoms of HPT, without measurement of PTH levels or an alternative explanation for hypercalcemia, is problematic [77]. The absence of symptoms alone is insufficient for diagnosing NFPC, as some functional PCs may be completely asymptomatic [61].

Similar to our NFPC Case 1, only three other cases have been reported with complete loss of PTH expression by immunohistochemistry [24, 34, 41]. In two of those cases, additional IHC panels were used to exclude alternative tumor types. In the third case, neither calcitonin nor non-medullary thyroid markers (TTF1, PAX8, TG) were assessed. That case also described only a metastasis of a previously diagnosed NFPC, without reporting the IHC profile of the primary tumor [24]. Additional details regarding the inclusion and exclusion of cases in our review are available in Online Resource 3.

None of the previously published NFPC cases have provided a comprehensive molecular characterization. Among the limited molecular studies available, one reported detection of retained PTH mRNA expression [40]. Posada-González et al. described an NFPC occurring in a patient with MEN2A, carrying a heterozygous *RET* mutation (Cys618Arg in exon 10), discovered through familial screening [37]. Khalil et al. identified an activating *PIK3CA* mutation and a frameshift *MEN1* mutation in an NFPC case. While the tumor showed partial parafibromin loss, the mutational status of *CDC73* was not reported [42]. To date, no NFPC case with confirmed *CDC73* mutation has been described. There is also inconsistent use of parafibromin immunohistochemistry in the literature. Most reports did not include it, and only two mentioned its application [42, 43].

Electron microscopy studies of NFPC revealed contradictory findings. Some reports describe an increased amount of cytoplasmic organelles, particularly prominent Golgi apparatus, along with numerous secretory granules, occasional lipid vacuoles, and glycogen—findings interpreted as evidence of impairment of hormone secretion leading to accumulation of secretory granules [13, 16]. In contrast, other studies observed abundant mitochondria but reduced numbers and size of the rough endoplasmic reticulum and Golgi apparatus, as well as cytoplasmic glycogen particles, few organelles, scarce secretory granules, and absence of lipid vacuoles [15, 19].

## Discussion

**Fig. 4:**
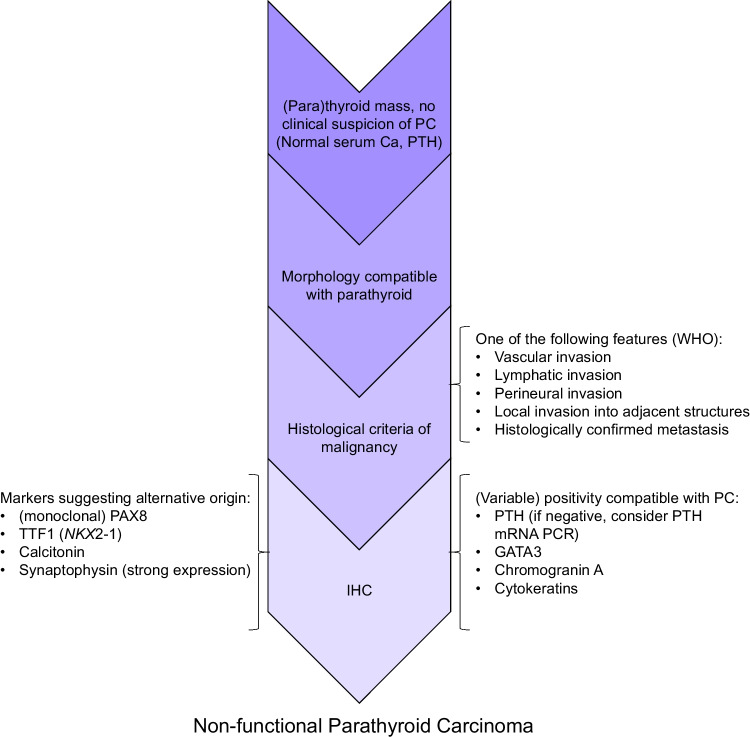
Diagnostic outline of non-functional parathyroid carcinoma (NFPC). Initial presentation of primary tumor typically involves a cervical mass without clinical or biochemical evidence of hyperparathyroidism (i.e., normal serum calcium and PTH levels). Morphology must be compatible with parathyroid tissue, followed by identification of histological features of malignancy as defined for parathyroid neoplasms by WHO criteria [74]. Immunohistochemistry (IHC) assists in determination of tissue origin. In addition to PTH, expression of GATA3, chromogranin A, and cytokeratins favors a parathyroid origin. In cases where PTH is absent by IHC, but other markers are consistent with parathyroid tissue, PTH mRNA PCR can aid in confirmation. IHC markers suggestive of an alternative tissue of origin include (monoclonal) PAX8 and TTF1 (*NKX2-1*), calcitonin, and strongly expressed synaptophysin [78, 79]. Ca calcium, IHC immunohistochemistry, PC parathyroid carcinoma, PTH parathyroid hormone, WHO World Health Organization

Non-functional parathyroid carcinoma (NFPC) is a particularly challenging diagnosis, especially in cases lacking PTH expression by immunohistochemistry. It is a rare entity, with approximately 50 cases reported to date. In the diagnostic workup, medullary and non-medullary thyroid carcinomas, paraganglioma, and other head and neck neoplasms must be carefully excluded. To assist with this, we provide a concise diagnostic outline (Fig. 4).

We now report two new cases of primary NFPC that were comprehensively studied at the molecular level.

In the first case, despite negative PTH immunostaining, the diagnosis was supported by low, but detectable PTH mRNA expression as previously described by Suganuma et al. [40].

In contrast, the second case showed patchy retained PTH staining, consistent with partial PTH translation [38]. Both tumors demonstrated massive whole chromosome losses, consistent with near-haploidization. Case 2 also showed subsequent endoreduplication/genome doubling, a phenomenon observed in other rare cancers such as oncocytic thyroid cancer (OCA) [74, 80–84]. We previously reported a case of functional PC case (*CDC73-*wildtype) with a near-haploid genome (NHG), but subsequent studies using exome or whole-genome sequencing have not identified similar findings [82, 85–87].

In oncocytic thyroid cancer, near-haploidisation has been linked to complex I mitochondrial inactivation and frequent complex I mitochondrial DNA mutations. While we did not assess mitochondrial DNA, electron microscopy in a previously reported NFPC case revealed abundant mitochondria, a hallmark of OCA [15].

Neither of the NFPC cases harbored *CDC73* mutations, which are common in conventional PC. Instead, we identified mutations in *MEN1* and *TERT* promoter (Case 1), and in *PTEN* as well as mutations in epigenetic-modifying genes *ARID1B*, *SETD2*, and *KDM5C* (Case 2). With the exception of *ARID1B* and *SETD2*, mutations in these genes have been previously reported in (functional) PC [87, 88]. The roles of *ARID1B and SETD2* have been recently investigated in tumors of other sites [89, 90].

Notably, *MEN1* and CDC73 mutations have been shown to be mutually exclusive in PC [87]. While *CDC73* mutations are common, *MEN1* mutations have also occasionally been reported [53, 55, 91]. The two NFPC cases presented here showed somatic mutations in *MEN1* and *PTEN*, respectively. Although mutations in these genes are typically associated with hereditary syndromes, neither patient displayed clinical features consistent with MEN1 or PTEN hamartoma tumor syndrome (Cowden syndrome).

Earlier molecular studies of NFPC are limited. Aside from reports of retained *PTH* mRNA expression mentioned above, Posada-González et al. described an NFPC occurring in a MEN2A patient with a germline heterozygous *RET* Cys618Arg mutation, and Khalil et al. reported a case with activating *PIK3CA* and frameshift *MEN1* mutations, along with partial parafibromin loss—but without *CDC73* mutation analysis [37, 42]. Across all NFPC cases in the literature, no *CDC73* mutations have been reported, and the parafibromin IHC has been inconsistently applied, mentioned in only two reports [42, 43].

Additionally, we identified a patient with Hyperparathyroidism-Jaw Tumor (HPT-JT) syndrome (germline *CDC73* mutation) and PC. Notably, this patient lost HPT by the time of metastatic disease 13 years after the resection of the primary functional tumor. The metastasis retained PTH expression by IHC. A similar clinical course has been reported once before in the literature [37]. As our study focused on primary NFPCs, we did not describe this patient in detail.

In both NFPC cases presented here, chromosome 11, where *PTH* is located, was among the lost chromosomes. While no somatic *PTH* mutations were found, several rare homozygous SNPs were detected, likely due to LOH and chromosomal loss. These homozygous SNPs have been discussed in the literature in the context of differential expression but do not fully explain the absence or reduction of PTH protein (Online Resource 2). Natural genetic variation at different gene loci is known to influence circulating PTH levels [92].

*PTH* promoter methylation was not investigated but could eventually contribute to silencing.

The *PTH* is located at 11p15.5, a region subject to parental genomic imprinting, involving genes such as *IGF2* and *H19* [93]. In imprinted regions, loss of the active allele can lead to gene silencing—a mechanism that may apply to *PTH* but remains speculative. Additionally, genes involved in PTH regulation, such as the calcium sensing receptor, could be affected, though this was not assessed.

Molecular pathways of PTH signalling, mutations occurring in congenital hypoparathyroidism, parathyroid adenoma, and functional PC were extensively studied [49, 59]. Despite these insights, the molecular mechanisms underlying reduced or absent *PTH* expression in NFPC remain unclear. Scarce data exist beyond case-level observations.

In our Case 1, the complete absence of PTH expression by IHC places it among only three other such NFPC cases previously reported [24, 34, 41]. It is important to acknowledge that many historic cases of NFPC (Table 1) were not subjected to PTH IHC, as this method could not be used prior to the invention and routine application of immunohistochemistry. In contrast, Case 2, like most NFPC cases in the literature, showed at least some degree of PTH immunoreactivity, despite normal PTH serum levels [18, 22, 23, 29, 31–33, 36, 38, 40, 42, 43, 46–48]. It is possible that a biologically active PTH molecule is synthesized but not secreted [13]. Alternatively, tumors may produce inactive forms of PTH, or abnormal protein processing or secretion may interfere with its detection. Another possibility is that currently available IHC and serum assays fail to detect altered PTH variants [13, 59]. Information regarding the specific epitopes targeted by commercial PTH IHC antibodies is largely unavailable due to proprietary restrictions. However, it is known that the first 27 amino acids at the N-terminus are essential for the biological activity of PTH [59].

In conclusion, we contribute two novel, well-characterized cases of primary NFPC. Our findings expand the molecular biology of NFPC and reinforce the need for integrated histopathological, immunohistochemical, and genomic evaluation in its diagnosis.

## Supplementary Information

Below is the link to the electronic supplementary material.

ESM 1 (40.4 KB DOCX)

ESM 2 (25.4 KB DOCX)

ESM 3 (28.8 KB DOCX)

## Data Availability

All data are available on request.
