# Supplementary material for: Diagnosing the silent: the molecular landscape of non-functional parathyroid carcinoma
Source: Virchows Arch. 2025 Aug 5;487(6):1247–63. doi: 10.1007/s00428-025-04193-4 (PMC12748313; doi:10.1007/s00428-025-04193-4)
Supplement: Supplementary file 1 — (40.4 KB DOCX) [file 428_2025_4193_MOESM1_ESM.docx]

## Materials and methods

Detailed methods are described below.

### Immunohistochemistry

Beside hematoxylin and eosin stain, the tumors were evaluated with immunohistochemical stains used in the routine diagnostics.

| Antibody | Clone | Company | Species |
| --- | --- | --- | --- |
| PTH | 3H9 | AbD Serotec | Mouse |
| SDHA | 2E3GC12FB2AE2 | ABCAM | Mouse |
| SDHB |  | Atlas | Rabbit |
| Parafibromin | 2h1 | Santa Cruz | Mouse |
| GATA3 | L50-823 | Cell Marque | Mouse |
| TTF1 | SPT24 | Monosan | Mouse |
| Synaptophysin (RTU) | DAK-SYNAP | Dako | Mouse |
| Chromogranin A | LK2H10 | Thermo Scientific | Mouse |
| Calcitonin (RTU) |  | Dako | Rabbit |
| S100 (RTU) |  | Dako | Rabbit |
| Keratine AE1/AE3 (RTU) | AE1/AE3 | Dako | Mouse |
| PAX8 |  | Protein Tech | Rabbit |
| Ki-67 | MIB-1 | Dako | Mouse |

**Supplementary Table 1:** The antibodies used in the routine diagnostics

### Molecular analysis

For molecular testing, total nucleic acid was isolated from formalin-fixed paraffin-embedded (FFPE) tissue after micro-dissection of serial hematoxylin-stained sections and selection of tumor tissue on basis of hematoxylin and eosin-stained diagnostic slides, including immunohistochemical stained slides in difficult cases. We performed Genome Wide-Imbalance/LOH/CNV analysis by sequencing 1,500 SNPs across all autosomes and the X chromosome using targeted NGS, data was analyzed as previously described [1,2]. When tumor cell percentage is sufficiently high - imbalances and LOH are identified from the SNP frequency patterns. Imbalances are then characterized by smaller amplitude changes when compared with LOH. Copy number detection by CNV analysis helps explain the mechanism behind the imbalances/LOH observed, being either chromosomal gains or losses. Imbalances in this test are scored when the alterations of the SNP profiles show a smaller amplitude in comparison with LOH which is scored below 0,25 and above 0,75 markings the plots. Imbalances often represent chromosomal copy number gains. Scoring of LOH can show genotypes A0 or 0B of individual SNPs. Genotypes AA or BB predict genome doubling/endoreduplication if multiple arms are affected and all heterozygous SNPs show extrapolated AABB genotypes. Copy neutral locus restricted LOH would show genotype AA or BB.

Additional mutational or fusion analysis was performed with diverse panels. The most recent information on used panels is publicly available on: <https://www.palga.nl/voor-pathologen/moleculaire-bepaling>. In the drop down menu choose LUMC to see the list of panels. There is also GW-LOH panel described above to be found.

### Cryo-sections

For case 1 (absent immunohistochemical PTH staining), fresh frozen tissue material was available. Frozen tissue of two functional parathyroid adenomas and a primary and metastatic functional parathyroid carcinoma were selected as positive controls. The frozen samples were withdrawn from the biobank LUMC with patient consent. Samples were handled according to the medical ethical guidelines described in the Code Proper Secondary Use of Human Tissue established by the Dutch Federation of Medical Sciences (www.federa.org). Five micron tissues sections were taken using a Leica CM3050S cryostat (Leica Microsystems B.V., Amsterdam, Netherlands), prior and after 20 µm sectioning for DNA or RNA isolation, and hematoxylin/eosin stained allowing microscopic examination for sufficient number of tumor cells in the samples. Three to ten 20 µm frozen sections were collected in a pre-cooled (-20^o^C) 1.5 mL micro vessel (Eppendorf Safe-Lock, Nederland B.V., Nijmegen, Netherlands, product no. 0030121503) and stored at -80^o^C until further use.

### *Cell culture*

The following human cell lines were used as a negative control for PTH expression: BHP 2-7, a derivative from TPC-1 [3] originating from a primary papillary thyroid cancer [4]. and immortalized retina pigment cells (RPE-1) [5].

Both cell lines were cultured in standard DMEM-F12 Nutrient Mixture (Ham) medium (Gibco, ThermoFisher Scientific, product no. 31330-38). The culture media were supplemented with 10% FBS (Sigma Life Science, Product no. F7524) and penicillin (50 U/mL), streptomycin (50 µg/mL) (Gibco, ThermoFisher Scientific, product No. 15140-122), diluted 1:200. Cell lines were maintained using standard culture conditions in protein-coated T75 flasks (Greiner Bio-One product no. 658940) and in a humidified atmosphere, 5% CO_2_ at 37 °C.

Cell lines were authenticate by a multiplex PCR (Cell ID GenePrint 10 system, Promega, product no. B9510). Data were compared with an online database (CelloSaurus, <https://web.expasy.org/cellosaurus/>, Swiss Institute of Bioinformatics) and both cell lines showed a 100% match. Cells tested for mycoplasma at regular intervals (in-house PCR method) and were no longer cultured than 20 passages.

### Cell harvesting

Medium from semi-confluent monolayer cell cultures (T75, Greiner Bio-One product no. 658940) was removed. BHP 2-7 cells attach firmly to the plastic and were first covered with HBSS, and replaced in the CO_2_ incubator at 37 °C. After 10 min 1x HBSS (Sigma Life Science, Product No. H4641) and the culture medium from RPE-1 were removed and the monolayers were covered with 3.0 mL 1x HBSS containing 0.1% trypsin (Gibco, ThermoFisher Scientific, product no. 15090-046) and 0.2 mM Na-EDTA (VWR Chemicals, product no. 20302.260), pH 7.2 and replaced in the cell incubator. Trypsin activity was blocked immediately after detachment by adding 1.5 mL foetal bovine serum (FBS, Merck/Sigma-Aldrich, product no. F7524). Next, cells were concentrated by centrifugation (500*g*, 5 min) washed once with HBSS (500*g*, 5 min) and finally resuspended in 1.0 mL HBSS and transferred to an 1.5 mL Safe-Lock tube (Eppendorf, product no. 0030121503).

### RNA isolation

Cells were concentrated by centrifugation once again (2000*g*, for 5 min). Frozen sections (see cryo-sectioning) or cell pellets were lysed and RNA was purified using the NucleoSpin RNA XS micro kit (Macherey-Nagel GmbH & Co. KG, Düren, BRD, product no. 740902.50) according to the manufacturer instructions. In short, cell pellets were lysed in buffer RA1 containing 2-β-mercaptoethanol and filtrated (11,000 x *g*, 1 min). Ethanol (70%) was added to adjust RNA binding conditions and the lysates were bound to NucleoSpin RNA columns and centrifuged (11,000 x *g*, 30 sec). Next the columns were desalted (11,000 x *g*, 1 min) and DNA was digested. In sequential steps the RNA was washed (11,000 x *g*, 30 sec, 30 sec and 1 min, respectively) and RNA was eluted in RNase-free water. RNA concentration was determined by spectrometry (N60 NanoPhotometer, ImPlen, Munich, Germany) and stored at -80^o^C until further use.

### cDNA synthesis

One microgram of total RNA was adjusted to a volume om 12.0 µL with RNase-free water. Samples were put at 60^o^C (heating block, Grant, QBT2, Grant Instruments, Cambridge, UK) and immediately cooled on ice after incubated for 15 min. Next 7.4 µL of the following reaction mixture was added to t he RNA: 0.5 µL RNAsin (10000 U, Promega, the Netherlands, product no. N2515), 0.5 µL oligodT (100 ng/µL) (PRIMER p (dT) 15, cDNA SYNTH., 8.0 nM, Roche Diagnostics, product no. 10814270001), 0.15 μL random d(n)6 primer (3 μg/μL), 2 µL dNTP’s) (50 units, ThermoFisher Scientific, product no. 48190-011) and 0.2 µL AMV-RT enzyme plus 4 µL RT buffer 5x, (1000 U, Roche Diagnostics, product no. 10109118001). Next, samples were put in a heating block at 42^o^C for 60 min followed by enzyme inactivation for 10 min at 65^o^C. Samples were cooled on ice, centrifuged (11,000 x *g*, 1 min) and stored at -20^o^C.

### RT-PCR

PCR was performed in a Real-Time PCR Detection System (CFX96, Bio-Rad, Veenendaal, the Netherlands) in 25 µl reactions comprising 10 ng DNA, iQ custom SYBR Green Supermix (Bio-Rad, product no. 172-5006 CUST) and 2.5 pmol PTH validated primers (Bio-Rad, Unique Assay ID:qHsaCID0006158). NADH-ubiquinone oxidoreductase subunit 1 (NDUFS1) validated primers (Bio-Rad, Unique Assay ID:qHsaCID0009325) were used as a positive control. The PCR conditions were 5 minutes at 95°C, 40 cycles of 30 seconds at 95°C, 30 seconds at 60°C, and 60 seconds at 72°C, with a final elongation step of 10 minutes at 72°C. After the final step, a melt curve was obtained to evaluate the quality of the PCR products. Data were analyzed using Bio-Rad CFX Maestro 2.3 software.

### DNA isolation

DNA was extracted using a NucleoSpin Tissue DNA isolation kit (item number 740901.50) (Macherey-Nagel GmbH & Co. KG, Düren, BRD) according to the manufacturer’s instructions. In short, tissue was digested overnight with proteinase K at 56^0^C. The following day DNA was purified by lysis followed by binding on a silica membrane, washing, drying and elution using highly purified water. DNA concentrations were determined using N60 NanoPhotometer (ImPlen, Munich Germany).

### DNA sequencing

For Sanger sequencing of the promotor region of exon 1, exon 1 and exon2/3 of PTH, 12 primer pairs (Merck, Darmstadt, Germany) with overlapping amplicons (primer sets 1 – 7: promotor region exon 1, exon 1 and exon 2 – 3, including the intronic region) were designed by the aid of Primer-Blast (National Centre for Biotechnology Information (NCBI), <https://www.ncbi.nlm.nih.gov/tools/primer-blast/>) using reference genome NC_000011.10. M13 tails were added to the primers for universal sequencing (Supplementary Table 2). The amplicons ranged between 400 and 513 bp. Each successive PCR product overlapped the previous amplicon by approximately 100 - 200 bp: exon 1 . PCR was performed in a Real-Time PCR Detection System (CFX96, Bio-Rad, Veenendaal, the Netherlands) in 25 µl reactions comprising 10 ng DNA, iQ Supermix (Bio-Rad) and 2.5 pmol primers, The PCR conditions were 5 minutes at 95°C, 40 cycles of 30 seconds at 95°C, 30 seconds at 62.5°C, and 60 seconds at 72°C, with a final elongation step of 10 minutes at 72°C. After the final step, a melt curve was obtained to evaluate the quality of the PCR products. Products ≥ 100 bp were purified using a MinElute 96 UF PCR Purification Kit (Qiagen) and a KNF Laboport solid PTFE vacuum pump (Merck). Purified PCR products were Sanger sequenced in both directions at GenomeScan (Leiden, Netherlands) and the DNA sequences were analyzed using Mutation Surveyor™ version 5.1.2 (Softgenetics, State College, PA). Variants were called against a FASTA GenBank reference sequence taken from chromosome 11 reference GRCh38.p14 primary assembly, and checked manually.

Supplementary Table 2. PTH Primers used for Sanger sequencing

| EXON | primer set |  | M13 | primers | Product length (bp) |
| --- | --- | --- | --- | --- | --- |
| 1 | 1 | FRW | *TGTAAAACGACGGCCAGT* | AGAATACTCTGCAGGAAAAACAGA |  |
|  |  | REV | *CAGGAAACAGCTATGACC* | GGGGTCTGCAGTCCAATTCAT | 501 |
|  |  |  |  |  |  |
|  | 2 | FRW | *TGTAAAACGACGGCCAGT* | AGCATTTTACCTTGAAGAAACAACA |  |
|  |  | REV | *CAGGAAACAGCTATGACC* | CCCCTTGTCAAGCCAAATGC | 404 |
|  |  |  |  |  |  |
|  | 3 | FRW | *TGTAAAACGACGGCCAGT* | GCAGACCCCTTAAATGGTGAC |  |
|  |  | REV | *CAGGAAACAGCTATGACC* | CCAGAAGTGACATGGATTGGTTG | 467 |
|  |  |  |  |  |  |
|  | 4 | FRW | *TGTAAAACGACGGCCAGT* | CCCTAAGGGATGATGAGAGCC |  |
|  |  | REV | *CAGGAAACAGCTATGACC* | AAATGGAGCCTGGAGCAACA | 402 |
|  |  |  |  |  |  |
|  | 5 | FRW | *TGTAAAACGACGGCCAGT* | AGCACTAACTGCATGCCTAAC |  |
|  |  | REV | *CAGGAAACAGCTATGACC* | CTTTGGTGGGAAGGCAAAGC | 490 |
|  |  |  |  |  |  |
|  | 6 | FRW | *TGTAAAACGACGGCCAGT* | CATTTGGCTTGACAAGGGGC |  |
|  |  | REV | *CAGGAAACAGCTATGACC* | GTTAGGCATGCAGTTAGTGCT | 468 |
|  |  |  |  |  |  |
|  | 7 | FRW | *TGTAAAACGACGGCCAGT* | ACCAATCCATGTCACTTCTGGT |  |
|  |  | REV | *CAGGAAACAGCTATGACC* | GAGCAGGCTCTCATCATCCC | 539 |
|  |  |  |  |  |  |
| EXON | primer set |  | M13 | primers | Product length (bp) |
| 2 + 3 | 8 | FRW | *TGTAAAACGACGGCCAGT* | GCCAGTGCCTAAGACAGTACA |  |
|  |  | REV | *CAGGAAACAGCTATGACC* | TTGCACTTTTAAACCTCTCTTCTAC | 511 |
|  |  |  |  |  |  |
|  | 9 | FRW | *TGTAAAACGACGGCCAGT* | CTTTCCTGAAACCAAAAGGACAACA |  |
|  |  | REV | *CAGGAAACAGCTATGACC* | AGTGTAGGGCAACAATACATGC | 513 |
|  |  |  |  |  |  |
|  | 10 | FRW | *TGTAAAACGACGGCCAGT* | ATCATGGCTAGTGATGGATTACA |  |
|  |  | REV | *CAGGAAACAGCTATGACC* | ACTGTTTTGCCTTGGAATTGGA | 502 |
|  |  |  |  |  |  |
|  | 11 | FRW | *TGTAAAACGACGGCCAGT* | CAGCTTCTTACGCAGCCATTC |  |
|  |  | REV | *CAGGAAACAGCTATGACC* | GTTGATTCCAAAAGCTTCTCGTG | 403 |
|  |  |  |  |  |  |
|  | 12 | FRW | *TGTAAAACGACGGCCAGT* | TCACTAAAAGGACAAGCAAAATGGA |  |
|  |  | REV | *CAGGAAACAGCTATGACC* | ACAATGTTTTCTTTTTCAGGTGAGT | 400 |

References

1. de Koster EJ, Corver WE, de Geus-Oei LF, Oyen WJG, Ruano D, Schepers A, Snel M, van Wezel T, Vriens D, Morreau H (2023) A clinically applicable molecular classification of oncocytic cell thyroid nodules. Endocr Relat Cancer 30(9).

<https://doi.org/10.1530/erc-23-0047>

2. Jentus MM, Bakker L, Verstegen M, Pelsma I, van Wezel T, Ruano D, Kapiteijn E, Crobach S, Biermasz N, Morreau H (2025) Chromosomal alteration patterns in PitNETs: massive losses in aggressive tumors. Endocr Relat Cancer 32(1).

<https://doi.org/10.1530/erc-24-0070>

3. Schweppe RE, Klopper JP, Korch C, Pugazhenthi U, Benezra M, Knauf JA, Fagin JA, Marlow LA, Copland JA, Smallridge RC, Haugen BR (2008) Deoxyribonucleic acid profiling analysis of 40 human thyroid cancer cell lines reveals cross-contamination resulting in cell line redundancy and misidentification. J Clin Endocrinol Metab 93(11):4331-4341.

<https://doi.org/10.1210/jc.2008-1102>

4. Ohta K, Pang XP, Berg L, Hershman JM (1997) Growth inhibition of new human thyroid carcinoma cell lines by activation of adenylate cyclase through the beta-adrenergic receptor. J Clin Endocrinol Metab 82(8):2633-2638.

<https://doi.org/10.1210/jcem.82.8.4136>

5. Bodnar AG, Ouellette M, Frolkis M, Holt SE, Chiu CP, Morin GB, Harley CB, Shay JW, Lichtsteiner S, Wright WE (1998) Extension of life-span by introduction of telomerase into normal human cells. Science 279(5349):349-352.

<https://doi.org/10.1126/science.279.5349.349>
