# Supplementary material for: Diagnosing the silent: the molecular landscape of non-functional parathyroid carcinoma
Source: Virchows Arch. 2025 Aug 5;487(6):1247–63. doi: 10.1007/s00428-025-04193-4 (PMC12748313; doi:10.1007/s00428-025-04193-4)
Supplement: Supplementary file 2 — (25.4 KB DOCX) [file 428_2025_4193_MOESM2_ESM.docx]

| **Case 1** |  |  |  |  |  |  |  |  |
| --- | --- | --- | --- | --- | --- | --- | --- | --- |
| **PTH** | **exon 1 + PROM** |  |  |  |  |  |  |  |
| **START** | **END** | **START CORRESPONDS TO POSITION 1** | **SNP ANALYSIS** | **SNP POSITION** | **INTRON** |  | **PMID/Reference** | **Notes from the reference text** |
| 13490999 | 13497999 | 13490999 | 4859A>G | 13495857 | rs3099597 | SNP | 34578986 | In table 1 – “no significant association with Vitamin D levels.” |
|  |  |  |  |  |  |  | 24367280, 23001124 | In the supplementary material, studies are not related with the topic. |
| 13490999 | 13497999 | 13490999 | 5349C>T | 13496347 | rs1380144 | SNP | <https://core.ac.uk/>  download/29051941.pdf | Mentioned in Table 5.1 |
| 13490999 | 13497999 | 13490999 | 6047T>G | 13497045 | rs2593570 | SNP | 34578986 | In table 1 – “no significant association with Vitamin D levels.” |
|  |  |  |  |  |  |  | 28079136 | In the supplementary material, observed SNP. |
|  |  |  |  |  |  |  | 23001124 | In the supplementary material, study is not related with the topic. |
|  |  |  |  |  |  |  |  |  |
| **PTH** | **exon 2-3** |  |  |  |  |  |  |  |
| **START** | **END** | **START CORRESPONDS TO POSITION 1** | **SNP ANALYSIS** | **SNP POSITION** | **INTRON** |  | **PMID/Reference** |  |
| 13490999 | 13497999 | 13490999 | 820A>C | 13491818 | rs307248 | SNP | 29969593 | Multiple mentions, association with kidney stone risk in the population of  West Bengal, India. |
| 13490999 | 13497999 | 13490999 | 933G>A | 13491931 | rs307247 | SNP | 34977236 | “The frequencies of PTH rs307247 genotypes were 10.8%, 35.8%, and  38.5% for A/A, A/G, and G/G in the CG compared to 12.6%, 31.5%, and 38.5%, respectively, in the postmenopausal osteoporosis group.” |
|  |  |  |  |  |  |  | **34063310** | **Table 1 reads: rs307247 - Low levels of PTH serum [citation - 18280230, see below].** |
|  |  |  |  |  |  |  | **18280230** | **“An association was identified between serum PTH (s-PTH) concentration and PTH SNPs rs307247 (p = 0.02) and rs307253 (p = 0.02). In both instances, heterozygotes had the highest s-PTH values (Table 6a). his could be explained under a recessive model, whereby individuals who possessed 1 or more copies of the variant allele had lower s-PTH levels (p-value 0.019 rs307247 and 0.018 rs307253). After correction for serum calcium and vitamin D levels, the p-values for rs307247 and rs307253 increased to 0.025 and 0.023 respectively.”** |
|  |  |  |  |  |  |  | 29969593 | Association with kidney stone risk in the population of West Bengal, India. |
|  |  |  |  |  |  |  | 36756694 | Association with tooth crown size. |
|  |  |  |  |  |  |  | 38922252 | “Was selected due to minor allele frequency, the study investigated  association of defects of enamel with polymorphisms in the vitamin D receptor and PTH.” |
|  |  |  |  |  |  |  | 23001124 | In the supplementary material, study is not related with the topic. |
| 13490999 | 13497999 | 13490999 | 1960C>T | 13492958 | rs177706 | ClinVar (benign) | 34578986 | In table 1 – “no significant association with Vitamin D levels.” |
| 13490999 | 13497999 | 13490999 | 2109_2112delATTT | 13493107 | rs1369686758 | SNP | <https://www.ncbi.nlm.nih.gov/>  snp/?term=rs1369686758 | No publications. |
|  |  |  |  |  |  |  |  |  |
|  |  |  |  |  |  |  |  |  |
| **Case 2** |  |  |  |  |  |  |  |  |
| **PTH** | **exon 1 + PROM** |  |  |  |  |  |  |  |
| **START** | **END** | **START CORRESPONDS TO POSITION 1** | **SNP ANALYSIS** | **SNP POSITION** | **INTRON** |  | **PMID/Reference** |  |
| 13490999 | 13497999 | 13490999 | 5349C>T | 13496347 | rs1380144 | SNP | <https://core.ac.uk/>  download/29051941.pdf | Mentioned in Table 5.1 |
|  |  |  |  |  |  |  |  |  |
| **PTH** | **exon 2-3** |  |  |  |  |  |  |  |
| **START** | **END** | **START CORRESPONDS TO POSITION 1** | **SNP ANALYSIS** | **SNP POSITION** | **INTRON** |  | **PMID/Reference** |  |
| 13490999 | 13497999 | 13490999 | 1718C>T | 13492716 | rs6254 | ClinVar (benign) | 36795755 | Used for family tree analysis. |
|  |  |  |  |  |  |  | 19557980 | “Patients with aortic stenosis were found to have a higher prevalence of the PTH AA genotype, while the VDR gene revealed a marginal, but statistically non-significant, association. The age and risk profile was similar in both groups.” |
|  |  |  |  |  |  |  | 18285546,  23401685,  20349051 | Only mention, no discussion. |
|  |  |  |  |  |  |  | 29969593 | Moderate association with kidney stone risk in the population of West Bengal, India. |
|  |  |  |  |  |  |  | 24903972 | “Schmitz et al. [*see above,* 19557980] revealed a positive association of  the A/A genotype of the rs6254 polymorphism within the PTH gene  with aortic stenosis whilst Gaudreault et al. did not replicate these results.” |
|  |  |  |  |  |  |  | 27756092 | “The present study supports the independent pathogenic effect of rs6254GA polymorphism on the development and severity of BMD complications in patients with asymptomatic but not normocalcemic HPT. The rs6254 PTH gene polymorphism has been extensively investigated and studies have found that it is linked to lower BMD and higher PTH levels in the general population, including young and  postmenopausal women [10024376] [11984699] [19690432] [18551993].” |
|  |  |  |  |  |  |  | 35195794 | "This study supports the significant role of rs1544410 and rs6254 polymorphisms and its significant allelic association with BMD either individually or in different combinations pertaining to osteoporosis susceptibility amongst  post-menopausal women from the south Indian population of Tamil Nadu." |
|  |  |  |  |  |  |  | 34977236 | “Genotyping cluster plots were reviewed manually, and three SNPs  (rs6254, rs10497900, and rs897083) showed poor cluster separation;  hence, they were excluded from the data set before further analysis.” |
|  |  |  |  |  |  |  | 36583005, 17319747,  29535823, 26549847,  29685792, 31597922,  24367280, 20886000,  32758128, 28099408,  28079136, 31434255,  23001124, 27082954,  29760388, 22022476 | Mention in the supplementary material. |
|  |  |  |  |  |  |  | 34578986 | In table 1 – “no significant association with Vitamin D levels.” |
|  |  |  |  |  |  |  | 22984424 | Association with BASFI/t for only one of the patient classifications (severe functional status in ankylosing spondylitis). |
|  |  |  |  |  |  |  | 18021008 | “SNP showing a significant sex × genotype interaction in the Belgian-Dutch population.” |
|  |  |  |  |  |  |  | 18551993 | “Part of the combined SNPs with  rs1799724-rs1800629-rs6254-rs6256-IL-1ra-rs2227956-rs1801197  was significantly associated with reduced bone mineral density.” |
|  |  |  |  |  |  |  | 18280230 | “Did not find evidence to associate polymorphism with bone density”. |
|  |  |  |  |  |  |  | 22390397 | "For serum PTH levels, we are not aware of any large GWA study, but in  a study by Tenne et al. (2008), six polymorphisms in the PTH gene  (rs307253, rs307247, rs6254, rs1459015, rs10500783 and rs10500784) had possible associations with the serum PTH level and these were included in our study. " No further mention/discussion. |
|  |  |  |  |  |  |  | <https://link.springer.com>  /article/10.1007/s13237-023-00456-0 | “In contrast, our previous study in the same population supports the  significant role of VDR-BsmI (rs1544410) and PTH-BstBI (rs6254)  polymorphisms and its significant allelic association with bone mineral  density either individually or in different combinations pertaining to osteoporosis susceptibility among postmenopausal women.” |
| 13490999 | 13497999 | 13490999 | 1872C>T | 13492870 | rs694 | ClinVar | **34063310** | **Table 1 reads: rs694 - Low levels of PTH serum [citation - 22722080, see below].** |
|  |  |  |  |  |  |  | **22722080** | **The c.-5-10 G > A (rs694) polymorphism detected in our patient has been described before and has no proven involvement**  **in the pathogenesis of hypoparathyroidism [citation 12905817], [citation 1672845]  Both cited works do not discuss PTH levels.** |
|  |  |  |  |  |  |  | 29969593 | “There were no significant differences between controls and kidney stone  patients in the distribution of either allele or genotype frequencies for rs694.” |
|  |  |  |  |  |  |  | 36756694 | “In this study, we observed strong associations in the multiple regression  model between polymorphisms in PTH (rs694 and rs307247) and the BL dimension of tooth crown size. “ |
|  |  |  |  |  |  |  | 38922252 | “SNPs in VDR and PTH were not associated with the etiology of human  developmental defects of enamel.” |
|  |  |  |  |  |  |  | 34578986 | In table 1 – “no significant association with Vitamin D levels.” |
|  |  |  |  |  |  |  | 26549847, 31597922, 28079136, 23001124, 24367280, 29334895,  18518984, 32758128, 25668207 | Mention in the supplementary material. |
