# Supplementary material for: Diagnosing the silent: the molecular landscape of non-functional parathyroid carcinoma
Source: Virchows Arch. 2025 Aug 5;487(6):1247–63. doi: 10.1007/s00428-025-04193-4 (PMC12748313; doi:10.1007/s00428-025-04193-4)
Supplement: Supplementary file 3 — (28.8 KB DOCX) [file 428_2025_4193_MOESM3_ESM.docx]

We excluded the patient described by Nomura et al: even though PC with negative PTH IHC was extensively described, this tumor was located in context of an adenoma, with marked increase in serum calcium and PTH [1]. We included the patient described by Ashkenazi – although there was no change in PTH levels after carcinoma surgery, during secondary exploration a concurrent adenoma was found resulting in PTH normalization [2]. The case reported by Krvavica was excluded due to the IHC profile (Thyroid Transcription Factor 1, TTF1 positivity) of tumor suggestive for a thyroid lesion [3]. TTF1 positivity excludes parathyroid as the tissue of origin [4-8]. However , “TTF1” refers to two different molecules [9]. The widely used “thyroid transcription factor 1” is encoded by *NKX2-1* (Protein NK2 homeobox 1) and historically referred to as “TTF1” or “TTF-1”. It possibly was confused with *TTF1* which encodes *Transcription Termination Factor 1*, which is expressed in majority of tissues and is not specific for thyroid and lung [9]. TTF1 (*NKX2-1*) mRNA is present in adult rat parathyroid tissue, but there is no expression in human normal, hyperplastic or adenomatous parathyroid tissue neither by RT-PCR nor by Southern blot independent of calcium concentrations [10]. Some medullary thyroid carcinomas indeed express TTF1 (*NKX2-1*) [4]. We excluded the patient reported by Messerer et al. – by normal calcium levels, the patient presented with PTH 3x above upper reference [11]. There is doubt whether the case reported by Guo et al. truly represents a (non-functional) parathyroid carcinoma [12]. The reported patient had a previous breast cancer — no description of histology or IHC profile — presented with multiple cervical lymph node metastases and a mass infiltrating the thyroid. The IHC profile (positive cytokeratin 19, synaptophysin, estrogen receptor; negative chromogranin A, calcitonin, thyroglobulin) can suit both – parathyroid and breast neoplasia (eventually with neuroendocrine differentiation) [13-15]. The given morphology does not give much support for one or another entity but strong expression of steroid receptor coactivator amplified in breast cancer-1 (*AIB1*, known as *NCOA3* – nuclear receptor coactivator 3) suggests the tumor more likely to be breast cancer [9].

1. Nomura T, Moriya T, Miyoshi K (2023) An extremely rare case of nonfunctioning parathyroid carcinoma occurring in a parathyroid adenoma. Med Mol Morphol 56(2):152-158.

<https://doi.org/10.1007/s00795-023-00350-3>

2. Ashkenazi D, Elmalah I, Rakover Y, Luboshitzky R (2006) Concurrent nonfunctioning parathyroid carcinoma and parathyroid adenoma. Am J Otolaryngol 27(3):204-206.

<https://doi.org/10.1016/j.amjoto.2005.01.006>

3. Krvavica A, Kovacić M, Baraka I, Rudić M (2011) Non-functioning parathyroid gland carcinoma: case report. Acta Clin Croat 50(2):233-237.

4. Mantovani G, Corbetta S, Romoli R, Alberti L, Beck-Peccoz P, Spada A (2001) Absence of thyroid transcription factor-1 expression in human parathyroid and pituitary glands. Mol Cell Endocrinol 182(1):13-17.

<https://doi.org/10.1016/s0303-7207(01)00555-x>

5. Shi Y, Brandler TC, Yee-Chang M, Cangiarella J, Wei X-J, Leung A, Szeto O, Deng F-M, Liu CZ, Simsir A, Sun W (2020) Application of GATA 3 and TTF-1 in differentiating parathyroid and thyroid nodules on cytology specimens. Diagn Cytopathol 48(2):128-137.

<https://doi.org/10.1002/dc.24338>

6. Uljanovs R, Sinkarevs S, Strumfs B, Vidusa L, Merkurjeva K, Strumfa I (2022) Immunohistochemical Profile of Parathyroid Tumours: A Comprehensive Review. Int J Mol Sciences 23(13):6981.

<https://doi.org/10.3390/ijms23136981>

7. Erdogan-Durmus S, Ramazanoglu SR, Barut HY (2023) Diagnostic significance of GATA 3, TTF-1, PTH, chromogranin expressions in parathyroid fine needle aspirations via immuonocytochemical method. Diagn Cytopathol 51(7):449-454.

<https://doi.org/10.1002/dc.25140>

8. WHO Classification of Tumours Editorial Board. Endocrine and neuroendocrine tumours [Internet]. Lyon (France): International Agency for Research on Cancer; 2022 [cited 2025 02 23]. (WHO classification of tumours series, 5th ed.; vol. 10). Available from: <https://tumourclassification.iarc.who.int/chapters/53>.

9. Pontén F, Jirström K, Uhlen M (2008) The Human Protein Atlas—a tool for pathology. J Pathol 216(4):387-393.

<https://doi.org/10.1002/path.2440>

10. Suzuki K, Kobayashi Y, Katoh R, Kohn LD, Kawaoi A (1998) Identification of thyroid transcription factor-1 in C cells and parathyroid cells. Endocrinology 139(6):3014-3017.

<https://doi.org/10.1210/endo.139.6.6126>

11. Messerer CL, Bugis SP, Baliski C, Wiseman SM (2006) Normocalcemic parathyroid carcinoma: an unusual clinical presentation. World J Surg Oncol 4(1):10.

<https://doi.org/10.1186/1477-7819-4-10>

12. Guo H, Mai R, Liu M, Peng H, Yang X, Wu M, Zhang G (2013) Nonfunctional parathyroid carcinoma after breast carcinoma. J Clin Oncol 31(9):e122-124.

<https://doi.org/10.1200/jco.2012.44.7227>

13. Bartek J, Bartkova J, Taylor-Papadimitriou J (1990) Keratin 19 expression in the adult and developing human mammary gland. Histochem J 22(10):537-544.

<https://doi.org/10.1007/BF01005976>

14. Haglund F, Ma R, Huss M, Sulaiman L, Lu M, Nilsson IL, Höög A, Juhlin CC, Hartman J, Larsson C (2012) Evidence of a functional estrogen receptor in parathyroid adenomas. J Clin Endocrinol Metab 97(12):4631-4639.

<https://doi.org/10.1210/jc.2012-2484>

15. Erickson LA, Mete O (2018) Immunohistochemistry in Diagnostic Parathyroid Pathology. Endocr Pathol 29(2):113-129.

<http://doi.org/10.1007/s12022-018-9527-6>
